# Annual trend of neonatal mortality and its underlying causes: population-based study – São Paulo State, Brazil, 2004–2013

**DOI:** 10.1186/s12887-021-02511-8

**Published:** 2021-01-26

**Authors:** Ruth Guinsburg, Adriana Sanudo, Carlos Roberto V Kiffer, Ana Sílvia S Marinonio, Daniela T Costa-Nobre, Kelsy N Areco, Mandira D Kawakami, Milton H Miyoshi, Paulo Bandiera-Paiva, Rita de Cássia X Balda, Tulio Konstantyner, Liliam CC Morais, Rosa MV Freitas, Mônica LP Teixeira, Bernadette Waldvogel, Maria Fernanda B Almeida

**Affiliations:** 1 Escola Paulista de Medicina – Universidade Federal de São Paulo, Rua Vicente Felix 77 apto 09, CEP 01410-020 São Paulo, SP Brazil; 2 Fundação Sistema Estadual de Análise de Dados, São Paulo, Brazil

**Keywords:** Infant newborn, Neonatal mortality, Infant, premature, Developing countries, Epidemiological studies

## Abstract

**Background:**

Population-based studies analyzing neonatal deaths in middle-income countries may contribute to design interventions to achieve the Sustainable Development Goals, established by United Nations. This study goal is to analyze the annual trend of neonatal mortality in São Paulo State, Brazil, over a 10-year period and its underlying causes and to identify maternal and neonatal characteristics at birth associated with neonatal mortality.

**Method:**

A population-based study of births and deaths from 0 to 27 days between 2004 and 2013 in São Paulo State, Brazil, was performed. The annual trend of neonatal mortality rate according to gestational age was analyzed by Poisson or by Negative Binomial Regression models. Basic causes of neonatal death were classified according to ICD-10. Association of maternal demographic variables (block 1), prenatal and delivery care variables (block 2), and neonatal characteristics at birth (block 3) with neonatal mortality was evaluated by Poisson regression analysis adjusted by year of birth.

**Results:**

Among 6,056,883 live births in São Paulo State during the study period, 48,309 died from 0 to 27 days (neonatal mortality rate: 8.0/1,000 live births). For the whole group and for infants with gestational age 22–27, 28–31, 32–36, 37–41 and ≥ 42 weeks, reduction of neonatal mortality rate was, respectively, 18 %, 15 %, 38 %, 53 %, 31 %, and 58 %. Median time until 50 % of deaths occurred was 3 days. Main basic causes of death were respiratory disorders (25 %), malformations (20 %), infections (17 %), and perinatal asphyxia (7 %). Variables independently associated with neonatal deaths were maternal schooling, prenatal care, parity, newborn sex, 1st minute Apgar, and malformations. Cesarean delivery, compared to vaginal, was protective against neonatal mortality for infants at 22–31 weeks, but it was a risk factor for those with 32–41 weeks.

**Conclusions:**

Despite the significant decrease in neonatal mortality rate over the 10-year period in São Paulo State, improved access to qualified health care is needed in order to avoid preventable neonatal deaths and increase survival of infants that need more complex levels of assistance.

## Background

Worldwide, deaths of children under five have dropped dramatically, with four million fewer deaths in 2015 compared to the year 2000 [1]. Such a reduction was based mainly in advances in the prevention and treatment of infectious diseases in the post-neonatal period and in children aged 1–4 years [2]. Although the neonatal mortality has also decreased during this period (36 per thousand in 1990 to 19 per thousand live births in 2014), this decline has not been as steep as that noted for post-neonatal mortality [3]. In 2016, in order to stimulate further decrease in neonatal mortality, the United Nations, within the Sustained Developmental Goals, called for an end to preventable deaths of newborns, with all countries aiming to reduce neonatal mortality to at least as low as 12 deaths per thousand live births [4].

In Brazil, the annual reduction in the under-5 mortality was 4 % between 2000 and 2015, with an estimated death rate in this age group of 16.9 per thousand live births in 2015 [5]. The neonatal mortality rate per thousand live births in the country fell from 16.7 to 2000 to 10.6 in 2011, corresponding to 64 % and 71 % of infant mortality, respectively [6]. Although the absolute reduction in neonatal mortality in Brazil has surpassed that noted in wealthier nations, the relative scale of this problem remains significant. If efforts are not made to close the gap in neonatal mortality, Brazil and other low- and middle-income countries will remain at a disadvantage [7].

In order to understand regional or national bottlenecks and design strategies to promote a further reduction in neonatal mortality, it is important to study why mothers and babies die. Using the three-delay model, problems may be related to delays in recognizing the need for care or in arriving at a health facility. When women and their babies do present to a medical center there may also be a delay in them receiving the right care [8]. It seems that in Brazil, where a public and accessible health care system does exist [9], but inequalities are pervasive throughout society [10], further improvement in maternal and newborn health outcomes will depend on the ability to understand and address the gap between coverage and quality [11].

São Paulo State is the world’s 28th most populous sub-national entity and the most populous sub-national entity in the Americas [12]. The state has the second-highest Human Development Index in the country (HDI: 0.826 in 2017) [13]. In São Paulo State, the neonatal mortality rate per thousand live births fell from 11.7 to 2000 to 7.9 in 2011, corresponding to 67 % and 68 % of the State infant mortality, respectively [6]. Despite these indicators, important social and economic inequalities occur throughout the State, as commonly seen in middle income countries [14]. Data available for the 645 municipalities of São Paulo State in 2010 show that HDI varied from 0.639 to 0.862 and infant mortality rate varied from 8.72 to 20.80 per thousand live births [13].

São Paulo is the only Brazilian state that developed, over decades, its own system of producing independent vital statistics, which manages to relate continuous data from the civil registry with epidemiological data originated from death and live birth certificates, producing comprehensive information that allows a consistent analysis of important health indicators [15]. In this context, the objective of this study was to analyze the annual trend of neonatal mortality in the state of São Paulo over a 10-year period and its underlying causes and to verify maternal demographic, prenatal and delivery care, and infants’ characteristics at birth associated with neonatal mortality.

## Method

A population-based study of live births and deaths from 0 to 27 days in 2004–2013 in the State of São Paulo, Southeast Brazil, was performed. Vital statistics database, originating from the Civil Registry of São Paulo State, was provided by the Foundation of the State System of Data Analysis (Fundação SEADE). Civil registry of births and deaths covers 99.7 and 99.8 % of these events in the State [16].

Two different sets of data were provided by Fundação SEADE [17]: (1) Data on all born alive infants during the study period retrieved from Certificates of Live Births (live birth registry); (2) Data on all infant deaths during the study period (infant death registry), retrieved from Death Certificates linked to Certificates of Live Births. The deterministic linkage of Death and Live Birth Certificates for the infant death registry was made by SEADE Foundation analyzing the files of all infants who died until 365 days with the respective registry of live births for each year, with the discordant cases being reviewed. To adapt both data sets to the study design, a database was created by integrating the infant death registry into the live birth registry, using the birth variables common to the two data sets, thus identifying those who died until 365 days in the live birth registry.

During the study period, infants were classified as alive if they survived the neonatal period or as neonatal deaths if death occurred between 0 and 27 days. Prematurity was defined if gestational age was < 37 weeks, as registered in the database. Infants were classified as being either extremely preterm (< 28 weeks), moderately preterm (28–31 weeks) or late preterm (32–36 weeks of gestation) [18]. Causes of neonatal death were classified according to ICD-10 codes as congenital malformations (codes Q), asphyxia (codes P20, P21, P24.0), respiratory disorders [codes P22, P23, P24 (except P24.0), P25 to P28], infections (codes P35 to P39), and others, according to the code written in the line of the Death Certificate dedicated to the basic cause of death [19].

For all infants in the database, the following information was collected: place of birth, municipality of birth, municipality of maternal residence, maternal age, marital status and maternal education, number of children born alive and dead in previous pregnancies, pregnancy duration in weeks, type of pregnancy (single or multiple), number of prenatal visits, type of delivery, time and date of birth, sex of the newborn, 1st and 5th minute Apgar score, birth weight, and presence of congenital malformation. For neonatal deaths, in addition to the items above, the following was collected: date of death, age of death, municipality where the death occurred, and causes mentioned in lines A, B, C, D of part I and part II of the Death Certificate. The basic cause of death was considered as the one present in the last fulfilled line of Part I.

All infants born in São Paulo State to mothers residing in the State in 2004–2013 were included in the analysis. Exclusion criteria were birthweight < 400 g and/or gestational age < 22 weeks. The annual trend of neonatal mortality rate according to gestational age was analyzed by Poisson Regression Model with robust variance for the whole study population and for infants ≥ 32 weeks of gestational age and by Negative Binomial Regression model with robust variance for those with 22–31 weeks. The different methods were applied because the assumption of Poisson regression is that the mean and the variance are equal, with an expected rate of deaths calculated according to the model fit. This assumption was satisfied for infants ≥ 32 weeks of gestational age, but it was violated for those < 32 weeks and a negative binomial regression model was performed.

Age at death was assessed for all neonatal deaths occurring during the 10-year study period using the Kaplan Meier curve. Hazard ratio (HR) of death during the neonatal period according to gestational age adjusted by year of birth was analyzed by Cox regression analysis.

Regarding variables associated with neonatal deaths, the maternal demographic variables (block 1), the prenatal and delivery care variables (block 2), and the neonatal characteristics at birth (block 3) were compared between two groups: newborn infants who died between 0 and 27 days after birth vs. those that survived for at least 27 days. Poisson regression analysis was applied in the three blocks, with backwards analysis for each block adjusted by year of birth. Joint analysis of the three blocks adjusted by year of birth led to the final model.

Statistical analysis was done using Stata/SE 15.1 (StataCorp, 2017. College Stattion, TX:StataCorp LLC). The study was approved by the Ethics Committee on Human Research of Escola Paulista de Medicina – Universidade Federal de São Paulo, and by the Board of Directors of Fundação SEADE.

## Results

**Fig. 1:**
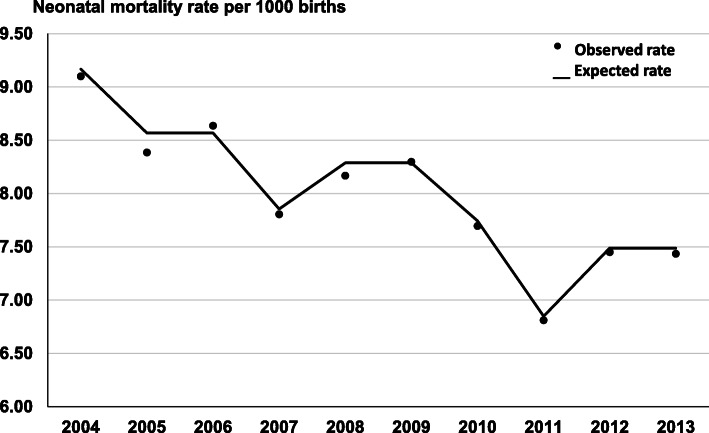
Observed and expected neonatal mortality rates per year adjusted by Poisson regression analysis, São Paulo State, Brazil, 2004–2013

From 2004 to 2013, there were 6,059,454 live births in São Paulo State, Brazil. Of these 2,571 (0.04 %) had a gestational age < 22 weeks or a birthweight < 400 g. Among the 6,056,883 births included in the study, 48,309 died from 0 to 27 days, with a neonatal mortality rate of 8.0 per thousand live births. Figure 1 shows the annual trend of neonatal mortality rate, adjusted by Poisson Regression analysis with robust variance, with an 18 % decrease from 2004 to 2013 (*p* < 0.001). For gestational ages of 22–27, 28–31, 32–36, 37–41 and ≥ 42 weeks, the reduction of neonatal mortality rate during the 10-year period was, respectively: 15 % (Negative Binomial Regression; *p* < 0.001); 38 % (Negative Binomial Regression; *p* < 0.001), 52 % (Poisson Regression; *p* < 0.001), 31 % (Poisson Regression; *p* < 0.001) and 58 % (Poisson Regression; *p* < 0.001) (Table 1).

**Table 1:** Neonatal mortality rate (95 % confidence interval) per 1000 births according to year of birth and gestational age, São Paulo State, Brazil

Birth Year	Total	22-27wk	28-31wk	32-36wk	37-41wk	≥ 42wk
**2004**	9.1 (8.9–9.3)	621 (601–640)	218 (206–230)	28.1 (26.5–29.9)	2.9 (2.7-3.0)	4.2 (2.4–6.9)
**2005**	8.4 (8.2–8.6)	610 (591–628)	201 (189–212)	24.4 (22.9–26.0)	2.5 (2.3–2.6)	4.7 (2.8–7.3)
**2006**	8.6 (8.4–8.9)	601 (581–621)	204 (192–216)	25.1 (23.5–26.7)	2.7 (2.5–2.8)	3.6 (1.8–6.4)
**2007**	7.8 (7.6-8.0)	534 (513–554)	173 (162–184)	22.5 (21.1–24.1)	2.6 (2.4–2.7)	5.7 (3.1–9.6)
**2008**	8.2 (7.9–8.4)	605 (585–625)	180 (169–192)	22.0 (20.6–23.6)	2.5 (2.4–2.7)	7.6 (4.4–12.1)
**2009**	8.3 (8.1–8.5)	584 (565–601)	180 (170–191)	22.5 (21.2–24.0)	2.5 (2.4–2.7)	6.2 (3.4–10.3)
**2010**	7.7 (7.5–7.9)	579 (560–598)	170 (159–180)	20.3 (19.1–21.8)	2.3 (2.2–2.4)	6.1 (3.2–10.4)
**2011**	6.8 (6.6-7.0)	508 (489–527)	142 (133–151)	17.8 (16.7–19.1)	2.0 (1.9–2.2)	1.8 (0.9–3.3)
**2012**	7.5 (7.2–7.7)	584 (566–602)	134 (126–143)	16.1 (15.1–17.2)	2.0 (1.9–2.1)	2.3 (1.5–3.3)
**2013**	7.4 (7.2–7.7)	517 (500–534)	128 (120–136)	13.4 (12.6–14.3)	2.0 (1.9–2.1)	2.3 (1.5–3.5)

**Fig. 2:**
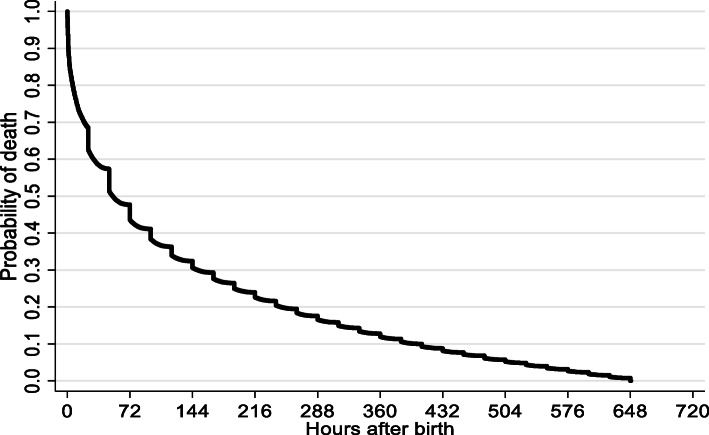
Age in hours at the time of death of the 48,309 infants who died from 0–27 days, according to Kaplan-Meier survival estimate, São Paulo State, Brazil, 2004–2013

Age in hours at death according to Kaplan Meier analysis is shown in Fig. 2. Median time until 50 % of deaths occurred for each gestational age group was: 1.39 days (95 %CI: 1.26–1.52) for 22–27 weeks (15,283 deaths); 3.29 days (95 %CI: 3.10–3.88) for 28–31 weeks (8,745 deaths); 3.00 days (95 %CI: 2.70-3.00) for 32–36 weeks (9,178 deaths); 3.00 days (95 %CI: 3.00–3.00) for 37-41weeks (12,638 deaths); and 1.40 days (95 %CI: 1.00–2.00) for ≥ 42 weeks (163 deaths). In the Cox Regression analysis adjusted by year of birth and using GA 37-41weeks as reference, the risk of neonatal death was: for GA 22–27 weeks - HR 366 (95 %CI: 358–375); for GA 28–31 weeks - HR 78 (95 %CI: 76–81); for GA 32–36 weeks - HR 8.9 (95 %CI: 8.6–9.1); and or GA ≥ 42 weeks - HR 1.5 (95 %CI: 1.3–1.8).

**Fig. 3:**
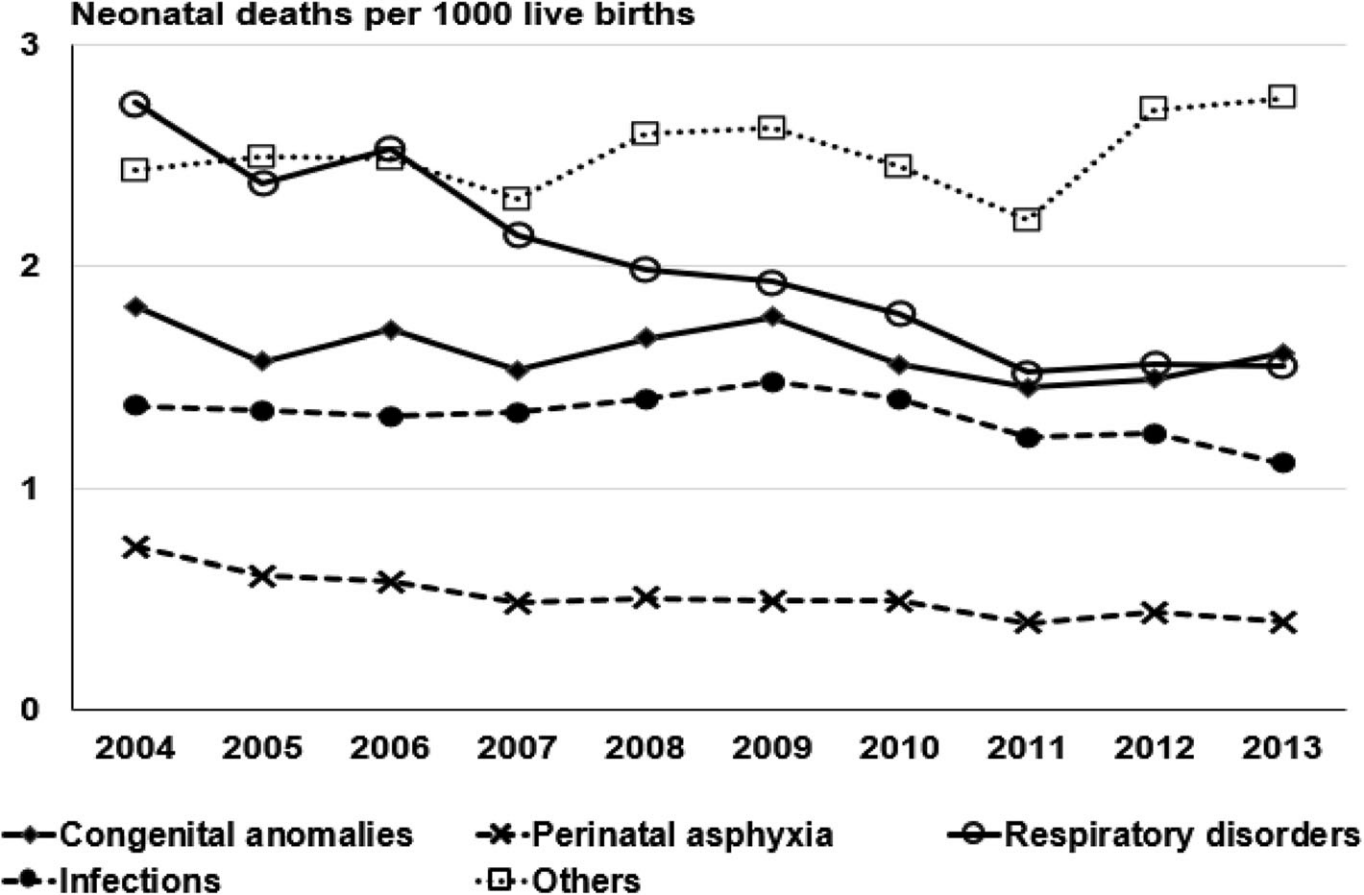
Neonatal deaths per thousand live births per year of birth, according to basic causes of death, São Paulo State, Brazil. 2004–2013

Most common causes of death for the whole study period included respiratory disorders in 25 %, congenital malformations in 20 %, infections in 17 %, perinatal asphyxia in 7 %, and other causes in 31 %. Figure 3 shows the neonatal deaths per thousand live births per year of birth, according to these underlying causes.

Information on maternal and neonatal variables was available for 96 % or more of the population, except for parity (92 %). Comparison of maternal demographic, prenatal and delivery care, and infants’ characteristics in neonates who died between 0 and 27 days after birth vs. those that survived for at least 27 days is shown in Table 2. Multivariate analysis of factors associated with neonatal death is shown in Table 3. The following variables, adjusted by year of birth, maternal age, marital status and multiple gestation, were shown to be risk factors for neonatal deaths: <12 years of maternal schooling; <7 pre-natal visits, male sex; 1st minute Apgar < 7 and presence of congenital malformations. Primiparity was a protective variable for neonatal death. There was an interaction between mode of delivery and gestational age associated with neonatal mortality. This interaction shows that cesarean delivery, compared to vaginal, was protective against mortality during the neonatal period for preterm infants at 22–31 weeks, but it was a risk factor for infants with 32–41 weeks (Table 4).

**Table 2:** Distribution of maternal and neonatal variables in the studied population according to neonatal death, São Paulo State, Brazil, 2004–2013

	Neonatal Death	
	**Yes (*n* = 48,309)**	**No (*n* = 6,056,883)**
Maternal age < 20 years	21 %	16 %
Maternal age ≥ 35 years	14 %	13 %
Unmarried mother	59 %	52 %
≤ 7 years of school	30 %	25 %
8–11 years of school	56 %	56 %
≥ 12 years of school	14 %	19 %
Primiparity	44 %	42 %
No prenatal visits	7 %	1 %
1–3 prenatal visits	19 %	4 %
4–6 prenatal visits	36 %	19 %
≥ 7 prenatal visits	37 %	75 %
Multiple gestation	10 %	2 %
Cesarean section	51 %	57 %
Non-hospital delivery	1 %	0.5 %
GA 22–27 weeks	33 %	0.2 %
GA 28–31 weeks	19 %	1 %
GA 32–36 weeks	20 %	8 %
GA 37–41 weeks	28 %	91 %
GA ≥ 42 weeks	0.4 %	0.8 %
Male	56 %	51 %
BW 400–999 g	38 %	0.2 %
BW 1000–1499 g	17 %	1 %
BW 1500–2499 g	20 %	8 %
BW 2500–2999 g	11 %	25 %
BW 3000–3999 g	13 %	62 %
BW ≥ 4000 g	1 %	4 %
1st minute Apgar 0–6	68 %	6 %
5th minute Apgar 0–6	40 %	1 %
Congenital malformations	27 %	1 %

**Table 3:** Variables associated with neonatal death adjusted by year of birth, maternal age, marital status, multiple gestation, mode of delivery and gestational age. São Paulo State, Brazil, 2004–2013

	*Incidence Rate Ratio*	95 % Confidence Interval
**Maternal schooling**		
≥ 12 years	reference	
< 7 years	1.20	1.13–1.26
8–11 years	1.11	1.06–1.16
**Parity**		
≥ 2	reference	
Primiparity	0.96	0.92–0.99
**Prenatal care**		
≥ 7 visits	reference	
0 visits	1.45	1.31–1.61
1–3 visits	1.43	1.35–1.54
4–6 visits	1.36	1.31–1.41
**Neonatal sex**		
Female	reference	
Male	1.09	1.05–1.13
**1st minute Apgar**		
7–10	reference	
0–2	20.14	18.85–21.51
3–6	6.38	6.17–6.60
**Congenital malformation**		
Absent	reference	
Present	15.10	14.28–15.98

**Table 4:** Variables associated with neonatal death: interaction of mode of delivery mode and gestational age. São Paulo State, Brazil, 2004–2013

	*Incidence Rate Ratio*	95 % Confidence Interval
**Mode of delivery**		
**C-section vs. vaginal**		
22–27 weeks	0.63	0.58–0.70
28–31 weeks	0.89	0.82–0.96
32–36 weeks	1.11	1.05–1.17
37–41 weeks	1.35	1.30–1.40
≥ 42 weeks	1.20	0.85–1.69
**Any GA vs. GA 37–41 weeks**		
**22–27 vs. 37–41 weeks**		
- Vaginal delivery	101.41	94.51–108.83
- Cesarean section	64.34	59.31–69.81
**28–31 vs. 37–41 weeks**		
- Vaginal delivery	28.02	26.08–30.12
- Cesarean section	24.87	23.35–26.49
**32–36 vs. 37–41 weeks**		
- Vaginal delivery	5.43	5.15–5.73
- Cesarean section	6.02	5.73–6.31
**≥ 42 vs. 37–41 weeks**		
- Vaginal delivery	1.34	1.04–1.74
- Cesarean section	1.61	1.28–2.03

Adjusted for year of birth, maternal age, marital status, years of study, multiple gestation, parity, prenatal care, neonatal sex, 1st minute Apgar score and congenital malformations

## Discussion

This population-based study shows a significant decrease in neonatal mortality rate over the 10-year period, mainly in neonates with 28–36 weeks. Throughout the study, 60 % of deaths occurred in the first 3 days after birth. Social indicators such as low maternal education and poor prenatal care, together with prematurity, perinatal asphyxia, and presence of congenital malformations, were associated with neonatal death in the State. Despite the fact that we cannot infer causality in the present study, delivery by cesarean, compared to vaginal, was protective against neonatal mortality for preterm infants at 22–31 weeks of gestational age, but it was a risk factor for those with 32–41 weeks.

Children face the highest risk of dying in their first month of life, at a global rate of 18 deaths per 1,000 live births. Globally, an estimated 2.5 million newborns died in the first month of life in 2018 – approximately 7,000 every day [3]. This population-based study shows that neonatal deaths decreased from 9.1 per thousand live births in 2004 (5,455 deaths; 15/day) to 7.4 per thousand in 2013 (4,543 deaths; 12/day) in São Paulo State. On one hand, the improvement in São Paulo State rates is noteworthy: in Latin America and Caribbean region as a whole and in Brazil in particular, the neonatal mortality decreased, respectively, from 13.1 to 14.7 per thousand live births in 2004 to 9.9 and 9.7 in 2014 [3]. On the other hand, if we compare our numbers with those retrieved from countries with a HDI similar to São Paulo State (0.82), such as Russia (0.82) and Argentina (0.83), the neonatal mortality rate per thousand live births was, in 2018, respectively, 3,0 and 6.0 [20], showing that despite improvements in São Paulo, the rates are exceedingly high. This paradoxical way of looking at the results may be the consequence of a lack of equilibrium between economic indicators and health care investments. As shown for middle income countries in general, the focus of public health system during the last 20 years was mainly aimed at developing the infrastructure for inpatient care of sick newborns, but these efforts were not followed by investments in quality of care [21].

The major reduction in neonatal mortality rates in the study period was seen in post-term (58 %), moderate preterm (52 %) and term (31 %) infants, and the worst performance occurred in those born with a gestational age < 28 weeks (15 % reduction). According to the World Health Organization, more than 80 % of premature births occur between 32 and 36 weeks of gestation. If essential care is offered promptly to these infants, it is possible that almost all of them will survive, even without the requirement for intensive care interventions [18]. In Brazil, in 2014, 86 % of premature births occurred in this gestational age range [6], indicating a great potential to prevent the death of preterm neonates. In fact, during the study period, among all preterm live births in São Paulo State, 85 % had a gestational age between 32 and 36 weeks and the decrease of the mortality rate was higher among them than the decrease observed in very preterm infants. Taken together with the reductions shown in the mortality rates of post-term and term infants from 2004 to 2013 and with the much smaller reduction in very preterm mortality rate in the same period, these observations suggest that essential newborn care was increasingly available throughout the State, but intensive care was either not largely available or did not have enough quality to ensure the survival of infants that required a more complex level of care. It seems that, even in the richest Brazilian State [12], further improvement in newborn health outcomes will depend on the ability to address the gap between coverage and quality [11].

In our study, the median time until 50 % of deaths occurred was 3 days for all infants and for all gestational age groups, except for infants with 22–27 and ≥ 42 weeks of gestations, in which 50 % of deaths occurred with 1.4 days after birth. Our results are also consistent with those reported in a systematic review of 22 studies in low- and medium-income countries, 62 % of the total neonatal deaths occurred during the first 3 days of life [22]. These findings are probably related to the suboptimal quality of care provided in health facilities. As highlighted by Sankar et al., facility-based care of neonates should be strengthened to improve the care of sick babies in the first few days of life, allocating more resources, including skilled manpower and finances, to existing facilities [22].

According to the “*Every Newborn”* action plan, three causes accounted for more than 80 % of neonatal mortality worldwide in 2012: complications of prematurity, intrapartum-related neonatal deaths (including birth asphyxia) and neonatal infections [23]. In our data, the main basic causes of death were respiratory disorders, that may be considered as a proxy for prematurity, infections, and perinatal asphyxia, with an important contribution of congenital malformations. The results indicate that São Paulo State, during the study period, faced a double challenge of dealing with neonatal deaths caused by preventable causes that prevail in medium and low-income countries, and, at the same time, dealing with deaths by congenital malformations that need a more complex level of care. Analysis of basic causes of deaths by year show that the State was able to decrease the rates associated with respiratory causes, probably in a parallel way to the large reduction of deaths in infants with 28–36 weeks of gestational age, and deaths caused by perinatal asphyxia. Deaths caused by infections and congenital malformations remained stable throughout the period. Again, the results suggest that, in São Paulo, during the study period, development of the infrastructure for inpatient care of sick newborns did occur, but investments in quality of care are still lacking [21].

In the multivariate analysis of variables associated with neonatal death in our population-based study, aside of confirming prematurity, perinatal asphyxia and congenital malformations as risk factors, maternal schooling, prenatal visits, parity, mode of delivery and sex were independently associated to neonatal mortality. There is an established causal and strong link between mothers’ education and child mortality. Gadikou et al. estimated that around 4 million fewer deaths of children under 5 years of age between 1970 and 2009 could be attributed to increased educational attainment in women of reproductive age [24]. In the State of Rio de Janeiro, Brazil, among 1,445,342 single pregnancy live births with birthweight ≥ 500 g and gestational age ≥ 22 weeks, less than 4 years of maternal education increased the chance of neonatal death in 25 % [25]. Our results, with a 20 % and 11 % increased death ratio associated, respectively, to maternal schooling < 7 years and 8–11 years reinforce the strong association of maternal education with not only access to health care, but access to qualified health care.

A similar view may be offered on the association between prenatal care and neonatal mortality. Data from 69 low- and middle-income countries from 1990 to 2013, including 752,635 observations for neonatal mortality, show that at least one antenatal care visit was associated with a reduced probability of neonatal mortality. Pregnant women who did not attend prenatal care were on average less educated and poorer than those who attended at least one visit [26]. In another study of 57 low- and middle-income countries, from 2005 to 2015, among 464,728 live births, there was 55 % lower risk of neonatal mortality among women who had the first antenatal care visit within the first trimester [27]. In our study having less than 4 prenatal visits was associated to 43–45 % increase incidence rate ratio of neonatal death. It is important to note that 25 % of mothers of the 48,309 infants that ultimately died in the neonatal period attended less than 4 prenatal visits during the study period. In 2014 and 2015, in Brazil, lack of access to prenatal care was twice more frequent in women with less than 4 years in school and in non-whites [28].

Variables associated to neonatal deaths highlight the social and economic inequalities prevalent in São Paulo State. This reinforced by our finding that primiparity was a protective variable against neonatal deaths. This result is similar to other findings in Brazil, where multiparity is associated with low socioeconomical level, inequalities in educational opportunities, and lack of access to family planning services, increasing the risk of these women to adverse obstetric outcomes [29].

According to the World Health Organization, “when medically justified, a cesarean section can prevent maternal and perinatal mortality and morbidity. There is no evidence, however, showing the benefits of the procedure for women or infants where it is not required” [30]. In fact, in our study, the delivery by cesarean section decreased the incidence rate ratio of neonatal deaths by 37 % and 11 % in infants of 22–27 and 28–31 weeks respectively, and increased this rate in more than 10 % in newborns with gestational age greater than 31 weeks. On one hand, the cesarean section rate is an important global indicator for measuring access to obstetric services [31], and it is possible that this route of delivery was indicated by complications that would increase the risk of mortality, such as fetal distress, placental abruption or chorioamnionitis. On the other hand, elective delivery, even at early term, is strongly associated with neonatal morbidity and mortality [32]. In a Brazilian study of 11,774,665 live births during 2014 to 2017, the Robson groups 1 to 4 accounted for 60 % of live births and 47 % of all cesarean sections, showing that health policies are needed to avoid the unnecessary cesarean sections [33]. Our data highlight once more that, in São Paulo State, the quality of care is a bigger barrier to reducing neonatal mortality than insufficient access [11].

The study has some limitations. The most important one is the use of secondary data, which may contain errors in records, underreporting and absence of clinical and care variables potentially associated with neonatal death. However, the database provided by SEADE Foundation manages to relate data from the civil registry with epidemiological data originated from death and live birth certificates, producing consistent information, and minimizing these problems [15]. Another limitation is the use of data updated until 2013, but the deterministic linkage of birth and death certificates for years 2014 on, that provide the database of our study, is still ongoing. We believe that the quality and consistency of the results presented in our study can be used to discuss time trends in neonatal mortality and its determinants and to design public policies in São Paulo State. Of course, the neonatal mortality data presented here is regional, but the discussion of causes and variables associated may be generalized to other regions and middle-income countries where economic development is not followed by social equity.

## Conclusions

This study showed a significant decrease in neonatal mortality rate in São Paulo State, Brazil, over the 10-year period, mainly in neonates with 28–36 weeks. Low maternal education and poor prenatal care, together with prematurity, perinatal asphyxia, and presence of congenital malformations, were associated with neonatal deaths in São Paulo State. Despite the fact that we cannot infer causality in the present study, cesarean delivery, compared to vaginal, was protective against neonatal mortality for very preterm infants, but it was a risk factor for those with gestational age ≥ 32 weeks. Efforts to reduce neonatal mortality in São Paulo State should focus on access to qualified health care.

## Data Availability

The complete database is available on request with the corresponding author.

## Abbreviations

SEADE Foundation: Fundação Sistema Estadual de Análise de Dados
ICD: International Classification of Diseases
HDI: Human Development Index
